# Updated mortality study of a cohort of asbestos textile workers

**DOI:** 10.1002/cam4.824

**Published:** 2016-07-25

**Authors:** Enrico Pira, Canzio Romano, Francesco S. Violante, Andrea Farioli, Giovanna Spatari, Carlo La Vecchia, Paolo Boffetta

**Affiliations:** ^1^ Department of Public Health Sciences and PediatricsTurin UniversityTurinItaly; ^2^ Department of Medical and Surgical Sciences (DIMEC)Bologna UniversityBolognaItaly; ^3^ Department of Environmental ScienceSafetyTerritoryFood and HealthMessina UniversityMessinaItaly; ^4^ Department of Clinical Sciences and Community HealthMilan UniversityMilanItaly; ^5^ Tisch Cancer InstituteIcahn School of Medicine at Mount SinaiNew YorkNew York

**Keywords:** Asbestos, cancer mortality, peritoneal cancer, pleural cancer

## Abstract

Limited information is available on risk of peritoneal mesothelioma after asbestos exposure, and in general on the risk of cancer after cessation of asbestos exposure. We updated to 2013 the follow‐up of a cohort of 1083 female and 894 male textile workers with heavy asbestos exposure (up to 100 fb/mL), often for short periods. A total of 1019 deaths were observed, corresponding to a standardized mortality ratio (SMR) of 1.68 (95% confidence interval [CI]: 1.57–1.78). SMRs were 29.1 (95% CI: 21.5–38.6) for peritoneal cancer, 2.96 (95% CI: 2.50–3.49) for lung cancer, 33.7 (95% CI: 25.7–43.4) for pleural cancer, and 3.03 (95% CI: 1.69–4.99) for ovarian cancer. For pleural and peritoneal cancer, there was no consistent pattern of risk in relation to time since last exposure, whereas for lung cancer there was an indication of a decline in risk after 25 years since last exposure. The findings of this unique cohort provide novel data for peritoneal cancer, indicating that – as for pleural cancer – the excess risk does not decline up to several decades after cessation of exposure.

## Introduction

The incidence of mesothelioma rises as function of the third or fourth power of time since first asbestos exposure (latency) 12, after taking into account time since cessation of exposure. The other determinants of mesothelioma incidence are average exposure to asbestos (linear relationship), and type of asbestos, with stronger potency of amphiboles than chrysotile 3. The influence of other time‐related aspects such as age at first exposure and duration of exposure appears to be largely or totally explained by latency 12. These models linking asbestos exposure to mesothelioma incidence have been developed and validated mainly on the basis of results for the pleural form of the disease, since most available cohort studies include a small number of peritoneal mesothelioma 2. Asbestos is also a cause of lung cancer, and cumulative asbestos exposure appears to be the main determinant of lung cancer incidence, together with the prevalence of tobacco smoking, because of the interaction between the two risk factors 3.

We reported the mortality follow‐up to 2004 of a cohort of heavily exposed asbestos textile workers employed between 1946 and 1984, in which we observed 315 cancer deaths compared to 153.9 expected (standardized mortality ratio [SMR] 2.11; 95% confidence interval [CI]: 1.89–2.35), including 39 deaths from peritoneal cancer, 36 deaths from pleural cancer, and 109 deaths from lung cancer 14, 15. In that analysis there was no difference in the SMR of pleural and peritoneal cancer between workers who had stopped exposure below age 30, and those who had continued exposure after age 40 15.

This cohort is unique since it contains a relatively large number of workers, in particular women, who experienced short‐term, high‐level exposure to asbestos. As discussed in detail in previous publications 14, 15, various types of asbestos were used in the factory, including crocidolite. Environmental exposure data were available between 1968 and 1977 14, 17. Average exposure levels as high as 100 fb/mL were measured in the opening and carding departments in the late 1960s; in the early 1970s average exposure levels were in the range 5–25 fb/mL, and in the late 1970s the average level was 2 fb/mL or less in all departments 14, 17.

The large number of deaths from peritoneal cancer is also an unusual finding. We decided therefore to update the follow‐up of this same cohort to 2013, aiming for separate analyses for pleural and peritoneal cancer, as well as lung cancer, with focus on the pattern of mortality following cessation of exposure.

## Materials and Methods

Detailed information on the cohort is provided in previous reports 14, 15. In brief, the cohort includes 1083 women and 894 men who had worked in an asbestos textile factory sited in Northern Italy, between 1946 and 1984. The main type of asbestos used in the plant was chrysotile, but crocidolite was also present, although the relative proportion of the two types of fibers over time is not precisely known. The current analysis includes 11 additional subjects as compared to the previous reports, who have been found to fulfill the inclusion criteria. For the present analyses, follow‐up was extended to November 2013; subjects were censored when they reached 85 years of age, because of the possibility of misclassification of cause of death in the elderly. Therefore, end of follow‐up was defined as date of death, date of last contact, date of 85^th^ birthday, or November 30, 2013, whichever occurred first.

We obtained employment data from personnel records at the factory, and ascertained vital status and causes of death through population registers and death certificates from local authorities. Subjects lost to follow‐up were censored at the date of last contact. Though complete information on occupational history was not collected, any available data on asbestos‐related jobs outside the factory were recorded, leading to the identification of 120 (6.1%) subjects with exposure to asbestos previous to the hire in the factory. Therefore, we conducted a sensitivity analysis excluding these subjects. We calculated several time‐related exposure variables, including time since first exposure (difference between date of hire and end of follow‐up), duration of employment (difference between date of hire and date of last employment), and time since cessation of exposure (difference between data of last employment and end of follow‐up).

We computed expected numbers of deaths using the national death rates during 1955‐1980 9, and the regional rates 1, 5 from 1981 onwards. Since national death rates were not available before 1955, the 1955–1959 death rates were applied to period 1946–1954. We computed the SMRs of selected cancers and of total mortality, as the ratio of observed and expected numbers of deaths. The corresponding 95% confidence intervals (CIs) were based on the Poisson distribution of observed deaths 4.

In addition, we fitted Poisson regression models to estimate mortality rate ratios for age at first employment, duration of employment, time since first employment, and time since last employment, after adjustment for sex and age 4. Age and the exposure variables were introduced in the regression models as time‐varying covariates; age was included as continuous variable to limit the number of parameters, and the addition of a term for age^2^ significantly increased the goodness of it of the models. To assess the presence of linear trends across levels of ordinal variables, we evaluated the Wald chi‐square statistic after fitting regression models including a linear term for the covariate of interest.

## Results

The present analyses include a total of 74,126 person‐years of observation (45,769 among women and 28,357 among men). The distribution of subjects and person‐years by demographic and exposure characteristics is shown in Table S1. Overall, 920 (46.5%) subjects were alive at the end of the follow‐up, 1019 (51.5%) had died and 38 (1.9%) emigrated or were lost to follow‐up. Cause of death was unknown for 48 (4.7%) of deceased subjects. Table 1 gives the SMRs in women, men and in both sexes combined. The SMR for all causes was 1.68 (95% CI: 1.57–1.78). Significant excesses mortality were observed for cancers of the peritoneum (SMR 9.1; 95% CI: 21.5–38.6), lung (SMR 2.96; 95% CI: 2.50–3.49), pleura (SMR 33.7; 95% CI: 25.7–43.4), and ovary (SMR 3.03; 95% CI: 1.69–4.99). The increased mortality was more pronounced in women than men. An increased in mortality from esophageal cancer, based on a small number of deaths, was present in women but not in men.

**Table 1:** Observed deaths from selected cancers, and corresponding standardized mortality ratios in a cohort of asbestos textile workers. Italy, 1946‐2013

		Women		Men		Overall	
Cause of death	ICD IX	Obs	SMR (95% CI)	Obs	SMR (95% CI)	Obs	SMR (95% CI)
Oral cavity and pharynx	140–149	0	0 (0–3.35)	9	2.03 (0.93–3.86)	9	1.64 (0.75–3.11)
Esophagus	150	4	6.45 (1.76–16.5)	3	0.89 (0.18–2.61)	7	1.76 (0.70–3.62)
Stomach	151	5	0.99 (0.32–2.32)	13	1.07 (0.57–1.83)	18	1.05 (0.62–1.66)
Colorectum	152–154, 159.0	9	0.93 (0.43–1.77)	24	1.73 (1.11–2.58)	33	1.40 (0.96–1.97)
Liver	155	4	0.96 (0.26–2.45)	8	1.12 (0.48–2.21)	12	1.06 (0.55–1.86)
Pancreas	157	1	0.23 (0.01–1.27)	8	1.47 (0.63–2.90)	9	0.92 (0.42–1.74)
Peritoneum	158	36	41.8 (29.3–57.9)	12	15.3 (7.88–27.0)	48	29.1 (21.5–38.6)
Larynx	161	0	0 (0–18.4)	8	1.95 (0.84–3.84)	8	1.84 (0.79–3.62)
Lung	162	42	5.29 (3.81–7.15)	101	2.51 (2.04–3.05)	143	2.96 (2.50–3.49)
Pleura	163	36	60.8 (42.6–84.2)	24	20.2 (13.0–30.1)	60	33.7 (25.7–43.4)
Breast (female)	174	16	0.86 (0.49–1.40)	–	–	16	0.86 (0.49–1.40)
Ovary	183	15	3.03 (1.69–4.99)	–	–	15	3.03 (1.69–4.99)
Prostate	185	–	–	7	0.94 (0.38–1.94)	7	0.94 (0.38–1.94)
Bladder	188	1	1.05 (0.03–5.85)	5	0.87 (0.28–2.03)	6	0.90 (0.33–1.96)
Kidney	189	3	2.46 (0.51–7.18)	1	0.35 (0.01–1.92)	4	0.97 (0.26–2.49)
Brain and central nervous system	191–192	3	1.20 (0.25–3.50)	5	1.59 (0.51–3.70)	8	1.42 (0.61–2.79)
Lympho‐hematopoietic malignancies	200–208	7	0.99 (0.40–2.04)	7	0.80 (0.32–1.65)	14	0.89 (0.48–1.49)
All cancers	140–239	210	2.41 (2.09–2.76)	254	1.87 (1.65–2.12)	464	2.08 (1.90–2.28)
All causes of death	1–999	401	1.84 (1.66–2.03)	618	1.58 (1.46–1.72)	1019	1.68 (1.57–1.78)
Person‐years			45769		28357		74126

Cause of death was unknown for 48 deceased subjects (4.7%). Obs, observed deaths; SMR, standardized mortality ratio; CI, confidence interval.

Table 2 gives the observed and expected numbers of deaths from peritoneal, lung, and pleural cancer according to time since last exposure, approximated by time since last employment. For pleural and peritoneal, there was no consistent pattern of SMRs, and no evidence of a decrease, in relation to time since last exposure. For lung cancer, the SMR declined after 25 years since last exposure. We repeated the latter analyses using different categories of time since last exposure (<15 years, 15 to <30 years and ≥30 years). The SMRs were higher in the category 15 to <30 years since last exposure than in the category ≥30 years (SMRs 45.1 and 26.8 for peritoneal cancer, 41.4 and 33.5 for pleural cancer, and 3.42 and 2.55 for lung cancer). SMRs according to time since first employment are reported in Table S2. SMRs for pleural cancers increased up to 40 years since first employment; in the case of ovarian cancer, the SMRs increased monotonically with time since first employment, although the number of deaths was small in several categories; no trend according to time since first employment was suggested for peritoneal and lung cancer.

**Table 2:** Observed deaths from peritoneal, pleural and lung cancer, and corresponding standardized mortality ratios, according to time since last employment in a cohort of asbestos workers. Italy, 1946–2013

Years since last employment	Peritoneal cancer		Lung cancer		Pleural cancer		Person‐years
	Obs	SMR (95% CI)	Obs	SMR (95% CI)	Obs	SMR (95% CI)	
<3^a^	1	8.40 (0.21–46.8)	7	1.85 (0.74–3.81)	1	14.9 (0.38–83.2)	15559
3–14	4	14.1 (3.83–36.0)	34	3.35 (2.32–4.68)	6	21.2 (7.78–46.2)	21517
15–24	17	50.1 (29.2–80.3)	41	3.67 (2.63–4.98)	14	33.7 (18.4–56.5)	15253
25–34	9	23.9 (10.9–45.4)	35	3.29 (2.29–4.58)	23	50.5 (32.0–75.9)	11710
≥35	17	32.3 (18.8–51.8)	26	2.08 (1.36–3.05)	16	28.8 (16.4–46.7)	10088

Obs, observed deaths; SMR, standardized mortality ratio; CI, confidence interval.

a Including current employment.

Table 3 shows the results of the multivariate Poisson regression analysis of risk of lung cancer, pleural cancer, and peritoneal cancer. Duration of employment was the exposure variable with the strongest association with risk of lung cancer, and a decrease in risk was suggested with time since last employment. In the case of pleural cancer, an association was found with time since first employment and time since last employment, but not with duration of employment. The results for peritoneal cancer suggest an association with duration of employment, as well as an increase in risk with time since first employment and time since last employment. Among workers employed for less than 1 year, there were 10 deaths from peritoneal cancer, 31 deaths from lung cancer, and 16 deaths from pleural cancer.

**Table 3:** Mortality rate ratio of peritoneal, pleural, and lung cancer in a cohort of asbestos textile workers. Estimates from Poisson regression models. Italy, 1946‐2013

Exposure variable	Peritoneal cancer			Lung cancer			Pleural cancer		
	*N*	MRR	(95% CI)	*N*	MRR	(95% CI)	*N*	MRR	(95% CI)
Age at first employment (years)									
<30	33	1.00	(Ref.)	45	1.00	(Ref.)	33	1.00	(Ref.)
≥30	15	0.47	(0.25–0.91)	98	1.25	(0.86–1.81)	27	0.79	(0.46–1.36)
Duration of employment (years)									
<1	10	1.00	(Ref.)	31	1.00	(Ref.)	16	1.00	(Ref.)
<5	15	1.49	(0.67–3.32)	34	1.33	(0.81–2.16)	21	1.39	(0.73–2.68)
5–9	8	1.34	(0.53–3.42)	27	1.78	(1.06–2.98)	10	1.15	(0.52–2.55)
≥10	15	2.42	(1.08–5.41)	51	2.95	(1.88–4.62)	13	1.43	(0.68–2.98)
*P trend*			0.05			<0.001			0.4
Time since first employment (years)									
<30	15	1.00	(Ref.)	72	1.00	(Ref.)	19	1.00	(Ref.)
30–44	24	1.67	(0.82–3.40)	57	0.83	(0.57–1.20)	33	2.48	(1.31–4.72)
≥45	9	1.82	(0.68–4.84)	14	0.59	(0.31–1.10)	8	2.13	(0.80–5.66)
*P trend*			0.8			0.09			0.03
Time since last employment (years)									
<15	5	1.00	(Ref.)	41	1.00	(Ref.)	7	1.00	(Ref.)
15–29	24	3.58	(1.34–9.54)	58	1.10	(0.72–1.67)	27	3.56	(1.53–8.31)
≥30	19	2.08	(0.73–5.89)	44	0.67	(0.42–1.06)	26	3.10	(1.26–7.67)
*P trend*			0.5			0.06			0.03

CI, confidence interval; MRR; mortality rate ratio, adjusted for sex and age; N, number of deaths; Ref., reference category; *P trend*, p‐value of test for linear trend.

The exclusion of 120 workers who experienced asbestos exposure before hire in the plant did not materially change the results: SMRs were 29.5 for peritoneal cancer (46 deaths), 2.97 for lung cancer (134 deaths) and 32.3 for pleural cancer (54 deaths). When we included also person‐years and deaths which occurred above age 85, resulting in a total of 74,535 person‐years of observation and 1097 deaths, the results were not materially changed. In particular, the SMRs were 28.4 (95% CI: 20.9–37.7) for peritoneal cancer, 32.6 (95% CI: 24.9–42.0) for pleural cancer, 2.95 (95% CI: 2.49–3.47) for lung cancer, and 3.17 (95% CI: 1.81–5.14) for ovarian cancer.

## Discussion

Using an updated follow‐up of this unique cohort of textile workers heavily exposed to asbestos, we were able to address the role of stopping exposure separately on pleural and peritoneal cancer. Both causes of death are related to asbestos exposure, but the latter has been studied less frequently than the former 2, and no data are available with respect to mortality from peritoneal cancer after stopping asbestos exposure.

The higher SMRs for pleural and peritoneal mesothelioma in women compared to men are probably attributable to the fact that women in the reference population have a lower rate of these neoplasms because of lower prevalence of past exposure to asbestos. Furthermore, in women, peritoneal cancer may be misclassified with ovarian cancer (Prat et al., 2015) and we found an increased mortality from the latter, though an order of magnitude smaller than peritoneal cancer. More in general, the use of mortality data, which refer to pleural and peritoneal cancer, might have resulted in misclassification of diagnosis of mesothelioma, and this might have differed according to time since cessation of exposure since during the follow‐up the International Classification of Diseases coding changed to the 10th Edition, which included a unique code for mesothelioma. We are currently retrieving the pathological samples of the members of the cohort who died from pleural and peritoneal cancers in order to indentify confirmed cases of mesothelioma, and we plan to conduct detailed analyses on different exposure variables restricted to the confirmed cases. Similarly, the higher SMR for lung cancer in women can be explained by lower rates in the reference population because of lower prevalence of tobacco smoking and exposure to occupational carcinogens.

The present results confirm those of previous studies 7, 11 on the persistence of excess pleural cancer risk after stopping asbestos exposure, and adds unique data on peritoneal cancer, indicating that also for this disease the excess risk does not level off up to 35 or more years since last exposure. This is consistent with the hypothesis of an important role of early asbestos exposure and of latency on subsequent mesothelioma risk, as it has been shown – mainly for the pleural form of the disease – in other studies 12; La Vecchia et al., 2000; 13; Frost, 2013).

In the case of lung cancer, the SMR appears to decrease 25 or more years since last exposure, although a possible effect of competitive mortality from mesothelioma might have played a role. With respect to the other exposure variables, the results of the multivariate analysis of mortality from lung cancer and pleural cancer are consistent with previous evidence that duration of exposure – and hence its derived variable, cumulative exposure, is the main determinant of risk of lung cancer, whereas latency (including time since last exposure) is the main determinant of risk of pleural cancer (Table S2; 3. Results on peritoneal mesothelioma are more difficult to interpret, and more conclusive evidence will be provided after the ongoing validation of diagnoses of mesothelioma from death certificates will be completed.

Table 4 compares the results on mesothelioma mortality with those of other cohort studies of asbestos textile workers: the higher proportion of deaths from mesothelioma in the present cohort is likely due to the use of crocidolite.

**Table 4:** Selected characteristics of cohort studies of asbestos textile workers

Reference	Country	Type of asbestos	% women	Total *N* deaths	N pleural meso. deaths	N peritoneal meso. deaths
McDonald et al. 19	USA	P Ch	0	1392	10	4
Peto et al. 20	UK	P Ch	0	1113	10	1
Dement et al. 21	USA	Ch	41	1259	2	0
Wang et al. 22	China	Ch	0	259	1	1
Wang et al. 23	China	Ch	32	285	1	2
This study	Italy	Mixed	48	1019	60	48

Ch, chrysotile; P Ch, predominantly chrysotile; meso, mesothelioma.

The choice to use regional reference rates was justified by the fact that region in which the plant is located has a higher mortality from mesothelioma and lung cancer than the country as a whole. This choice might have resulted in some underestimate of the risk of asbestos‐related diseases since a relatively large proportion of the reference population was also exposed to asbestos. However, regional rates were not available for the first part of the follow‐up, and we used national rates.

Among the other neoplastic causes of death, it is worth considering the nonstatistically significant increased SMRs for cancers of the head and neck, the esophagus and the colorectum. While an association between asbestos exposure and head and neck cancer (in particular laryngeal cancer) has been reported in several populations 8, and the lack of statistical significance in this cohort might be due to the rarity of these malignancies in women (all deaths occurred among men), the data on risk of esophageal and colorectal cancer among asbestos workers are more controversial 8. The lack of consistency of the results between the female and the male components of the cohorts detracts from a causal interpretation of the findings for these two neoplasms.

Strengths of the study include on the unique exposure circumstances of this cohort, the long follow‐up, and the low proportion of cohort members lost to follow‐up. Limitations include the lack of individual exposure data, reliance of death certificates for outcome assessment – which can be particularly problematic in case of pleural and peritoneal cancer 18, and lack of information on potential confounders and effect modifiers, such as tobacco smoking in the case of lung cancer, and alcohol drinking for oral and pharyngeal cancer. As discussed above, we are conducting a validation of the diagnoses of pleural and peritoneal cancer, to increase the specificity of the assessment of outcome, and to repeat the analyses according to time‐related variables on a subset of confirmed deaths from peritoneal and pleural mesothelioma. Furthermore, we did not have complete information on employment of cohort members on other industries entailing asbestos exposure, which may results in misclassification of time‐related exposure variables, such as time since last exposure. In this respect, age at first exposure might be a good indicator of the effect of early exposure because it is less correlated with the other time‐related variables: an effect of age at first exposure is present for peritoneal and pleural cancer, but not for lung cancer.

In conclusion, the findings of this cohort provide novel evidence for peritoneal cancer, indicating that – as for pleural cancer – the excess risk does not decline up to several decades after cessation of exposure.

## Supporting information


**Table S1**. Selected characteristics of the cohort.
**Table S2**. Observed deaths (O) from peritoneal, pleural, lung and ovarian cancer, and corresponding standardized mortality ratios (SMR), according to time since first employment in a cohort of asbestos workers. Italy, 1946‐2013.
