# Supplementary material for: Updated mortality study of a cohort of asbestos textile workers
Source: Cancer Med. 2016 Jul 25;5(9):2623–8. doi: 10.1002/cam4.824 (PMC5055168; doi:10.1002/cam4.824)
Supplement: Supplementary file 1 — Table S1. Selected characteristics of the cohort. Table S2. Observed deaths (O) from peritoneal, pleural, lung and ovarian cancer, and corresponding standardized mortality ratios (SMR), according to time since first employment in a cohort of asbestos workers. Italy, 1946‐2013. [file CAM4-5-2623-s001.docx]

Supplementary Table 1. Selected characteristics of the cohort.

|  | **Subjects** | **Person-years** |
| --- | --- | --- |
|  |  |  |
| **Overall cohort** | 1977 | 74126 |
|  |  |  |
| **Sex** |  |  |
| Women | 1083 | 45769 |
| Men | 894 | 28357 |
|  |  |  |
| **Exposure characteristics** |  |  |
| **Duration of exposure** |  |  |
| <1 | 691 | 28363 |
| 1-4 | 543 | 22734 |
| 5-9 | 327 | 12193 |
| ≥10 | 416 | 10837 |
|  |  |  |
| **Age at first exposure** |  |  |
| <25 | 838 | 37564 |
| 25-34 | 506 | 19298 |
| ≥35 | 633 | 17265 |
|  |  |  |
| **Period at first exposure** |  |  |
| Before 1955 | 401 | 14446 |
| 1955-1964 | 567 | 24084 |
| 1965-1971 | 816 | 28967 |
| 1972 or later | 193 | 6629 |

Supplementary Table 2. Observed deaths (O) from peritoneal, pleural, lung and ovarian cancer, and corresponding standardized mortality ratios (SMR), according to time since first employment in a cohort of asbestos workers. Italy, 1946-2013.

| **Years since first employment** | **Peritoneal cancer** | | **Pleural cancer** | | **Lung cancer** | | **Ovarian cancer** | | **Person-years** |
| --- | --- | --- | --- | --- | --- | --- | --- | --- | --- |
|  | O | SMR | O | SMR | O | SMR | O | SMR |  |
| <20 | 0 | 0 | 6 | 22.2 | 25 | 2.12 | 1 | 1.51 | 36205 |
| 20-29 | 14 | 40.0 | 16 | 36.4 | 46 | 3.88 | 2 | 1.98 | 16031 |
| 30-39 | 17 | 36.2 | 23 | 41.1 | 44 | 3.31 | 4 | 2.70 | 12907 |
| 40+ | 17 | 33.3 | 15 | 29.4 | 28 | 2.47 | 8 | 4.44 | 8984 |

Deaths and person-years occurring at age ≥85 years are excluded.
